# Pre-marital sex and its association with peer pressure and watching pornography among young individuals in Ethiopia: a systematic review and meta-analysis

**DOI:** 10.1038/s41598-022-13448-y

**Published:** 2022-06-10

**Authors:** Yitayish Damtie, Nigus Cherie, Habtamu Fentaw, Bereket Kefale, Elsabeth Addisu, Melaku Yalew, Mastewal Arefaynie, Metadel Adane, Bezawit Adane, Assefa Andargie Kassa, Aregash Abebayehu, Fanos Yeshanew Ayele

**Affiliations:** 1 Department of Reproductive and Family Health, School of Public Health, College of Medicine and Health Sciences, Wollo University, Dessie, Ethiopia; 2 Master of Public Health, School of Public Health, College of Medicine and Health Sciences, Wollo University, Dessie, Ethiopia; 3 Department of Environmental Health, College of Medicine and Health Sciences, Wollo University, Dessie, Ethiopia; 4 Department of Epidemiology and Biostatistics, School of Public Health, College of Medicine and Health Sciences, Wollo University, Dessie, Ethiopia; 5 Department of Public Health Nutrition, School of Public Health, College of Medicine and Health Sciences, Wollo University, Dessie, Ethiopia

**Keywords:** Medical research, Risk factors

## Abstract

There is no national representative estimate on pre-marital sex and its association with peer pressure and watching pornography among young individuals in Ethiopia. So, this study aimed to estimate the pooled prevalence of pre-marital sex and its association with peer pressure and watching pornography among young individuals in Ethiopia. A comprehensive search of international databases including CINAHL, Google Scholar, Cochrane Library, PubMed, HINARI, and Global Health was carried out to estimate the pooled prevalence of pre-marital sex and its association with peer pressure and watching pornography among young individuals in Ethiopia. The data were analyzed using STATA/SE version-14. The random-effect model was used to estimate the effect size and I-squared statistics and Egger's test were used to assess the heterogeneity publication bias respectively. A total of thirty-two studies with 18,354 study subjects were included in this meta-analysis. The pooled prevalence of premarital sex among young in Ethiopia was 33.59% [95% CI (29.09, 38.09)]. There was significant heterogeneity among the included articles (I^2^ = 97.9, *p* = 0.000). Young individuals who experienced peer pressure were three times more likely to practice premarital sex compared to their counterparts [OR = 2.90, 95%, CI (1.01, 8.31)]. As the crude analysis result indicated, there was a significant association between watching pornography (sex movies) and premarital sexual practice [OR = 3.41, 95% CI (1.99, 5.84)]. However, after doing trim-and-fill analysis, the publication-bias adjusted OR indicates the absence of significant association between watching pornography and premarital sex [OR = 1.23, 95% CI (0.69, 1.76)]. The proportion of premarital sex among young individuals in Ethiopia remains high. Peer pressure had a statistically significant association with premarital sexual practice. However, the publication-bias adjusted OR indicates the absence of a significant association between watching pornography and premarital sex. Peer counseling services, sex education, and behavioral change communications should be strengthened to address factors associated with pre-marital sexual practices.

World Health Organization (WHO) defines young as a person aged from 10 to 24 years old^1^. Young individuals are a large segment of the population comprising 1.8 billion (27%) world population of which 90% of them live in developing countries^2,3^. Young is the stage of transition from childhood to adulthood and is characterized by a spurt of physical, biological, emotional, social, mental, and psychosexual developments which are accompanied by either positive or negative behaviors depending on the environment that the child is brought up^4^. Many important life events and risky behaviors including pre-marital sexual practices start during this stage^5^.

Premarital sex is penetrative sexual intercourse performed before a formal marriage^6^. The rate of premarital sexual practice among young’s varies from country to country. It is 4.3% among university students in Turkey^7^, 18.1% in China^8^, and 47.5% among youth in Southern Iran^9^. It is quite common in Africa compared to other continents. A recent study in Nigeria revealed that the rate of premarital sex among university students was 45.8%^10^ and it was 70.4% and 74% in Tanzania and Uganda respectively^11,12^. In Ethiopia the prevalence ranged from 17.5% to 71.9%^13,14^.

Premarital sexual debut among youth is mostly unprotected and therefore, exposes them to the risk of Human Immune Virus/ Acquired Immunodeficiency Syndrome (HIV/AIDS) and other sexual transmitted infections (STIs)^8,15,16^. It is also associated with a greater risk of unwanted pregnancy and unsafe abortion leading to maternal morbidity mortality^17–19^. Moreover, it also leads to, loss of family support, self-respect, and depression^17^. Premarital sex is affected by several factors such as socio-demographic characteristics of young (age, sex, and level of education)^20–22^, peer pressure^8,17,22–24^, substance use^8,17^, having pocket money, discussion with parents about sexual issues^20^, religiosity^21^ and watching pornography^8,24^.

The premarital sexual practice among young individuals was well studied in different parts of Ethiopia^5,13,14,25–53^ and a systematic review and meta-analysis study was also done in 2019 even if it is not published^54^. But, the study considered only twenty-four studies in the review and couldn't assess the association between peer pressure and premarital sex. There is no national representative estimate on the association between peer pressure and watching pornography with premarital sex in Ethiopia. So, this study aimed to estimate the pooled prevalence of pre-marital sex and its association with peer pressure and watching pornography among young individuals in Ethiopia. Peer pressure and watching pornography are clinically important and frequently mentioned factors affecting premarital sex although they have inconsistent findings across the included articles. Sexual and reproductive health of the young is one of the primary intervention areas of Sustainable Developmental Goals SDGs)^55^. In line with this, the Ethiopian government also develop different strategies targeting adolescents and youth reproductive health that has to be achieved at the end of 2020^56^. So, this meta-analysis will generate crucial evidence for program planners and policymakers to design evidence-based interventions to decrease premarital sex.

## Materials and methods

### Searching strategy

This meta-analysis followed the Preferred Reporting Items for Systematic Reviews and Meta-Analyses (PRISMA-2009) guideline^57^ (see Supplementary File 1) and the protocol for this study was registered and published at PROSPERO, an international prospective register of systematic reviews with the identification number of CRD42020179502. A comprehensive search of international databases including CINAHL, Google Scholar, Cochrane Library, PubMed, HINARI, and Global Health was carried out to estimate the pooled prevalence of pre-marital sex and its association with peer pressure and watching pornography among young individuals in Ethiopia. The search was conducted from February 1up to March 30, 2021by three reviewers (YD, NC, and HF) independently and articles published from 2000 up to March 30, 2021were included in this systematic review and meta-analysis.

In the searching process, studies were identified using the following key terms: "proportion", "prevalence", "incidence", "magnitude", "premarital sex", "premarital sexual practice",“ premarital sexual debut”, “premarital sexual intercourse”, "predictors", "risk factors", "determinants", "factors", "associated factors", "students", " adolescents", "youths", " young ", "Ethiopia" using Boolean operators "AND" and "OR"(see Supplementary File 2).

### Eligibility criteria

#### Inclusion criteria

- **Population**: This meta-analysis includes studies conducted among young individuals of male, female, or both sexes,
- **Exposure**: young individuals who experienced peer pressure and watch pornography (sex films),
- **Comparison**: young individuals who didn’t experience peer pressure and didn't watch pornography (sex films),
- **Outcome**: Studies assessed premarital sex as a primary outcome,
- **Study setting**: All community and institution-based studies,
- **Study design**: Studies with a cross-sectional study design,
- **Publication**: Published and unpublished articles,
- **Country**: Studies done in Ethiopia,
- **Language**: studies written in the English language,
- **Time frame**: studies published from 2000 up to March 30, 2021were included in this study.

#### Exclusion criteria

- Those studies with the absence of full texts were excluded.

### Outcome measurement

This meta-analysis measured two key outcomes. The primary outcome of the study was to estimate the pooled prevalence of premarital sex which was computed from the individual article by dividing the number of young individuals practicing premarital sex to the total sample size multiplied by 100. The second outcome was the association between peer pressure and watching pornography with premarital sex. For the second outcome, the association between explanatory variables (peer pressure and watching pornography) and premarital sex was determined in the form of the log odds ratio.

### Data extraction and quality assessment

All articles gathered from different databases were exported to the endnote reference manager, and duplicates were identified and removed. The remaining articles were screened based on their title and abstract and evaluated in the context of the inclusion criteria by three independent reviewers (MY, MA, and MA). Then Joanna Briggs Institution (JBI) quality assessment tool was used to appraise the qualities of the screened articles and those articles scoring 50% and more were included in the analysis^58,59^. In this meta-analysis, all of the included studies scored 50% and more thus, all are included in the review. Two authors (BK and BA) independently assessed the quality of the studies and the mean score was taken to manage the different results obtained from both reviewers.

All the necessary data were extracted using a Microsoft Excel sheet. The data extraction sheet includes; the name of the author, study area, region, publication year, year of study, study design, study setting, the prevalence of premarital sex, response rate, sample size, number of young individuals practicing premarital sex, residence, sex and frequencies of watching pornography film and peer pressure in the form of a two by two tables. Four independent authors (EA, AAK, FY, and AA) extract all the data and the discrepancy between reviewers was resolved through consensus.

### Data analysis

All the extracted data were exported to STATA version-14 for further analysis. The random effect model at a *p* value < 0.05 was used to compute the pooled prevalence of premarital sexual practice among students in Ethiopia^60^. In addition, the association between peer pressure and watching pornography with premarital sexual practice was statistically estimated using pooled odds ratios with 95% CI.

The I^2^ statistic was used to assess the heterogeneity between the included studies and I^2^ tests at a value of 25%, 50%, and 75% were considered as low, medium, and high heterogeneity. Subgroup analysis and univariate meta-regression were carried out to identify the source of variations among studies that exhibited severe heterogeneity. Moreover, publication bias was assessed using the funnel plot and egger's test. A *p* value of less than 0.05 in the Egger regression test is considered as the presence of statistically significant publication bias^61^.

## Results

### Study selection

**Figure 1:**
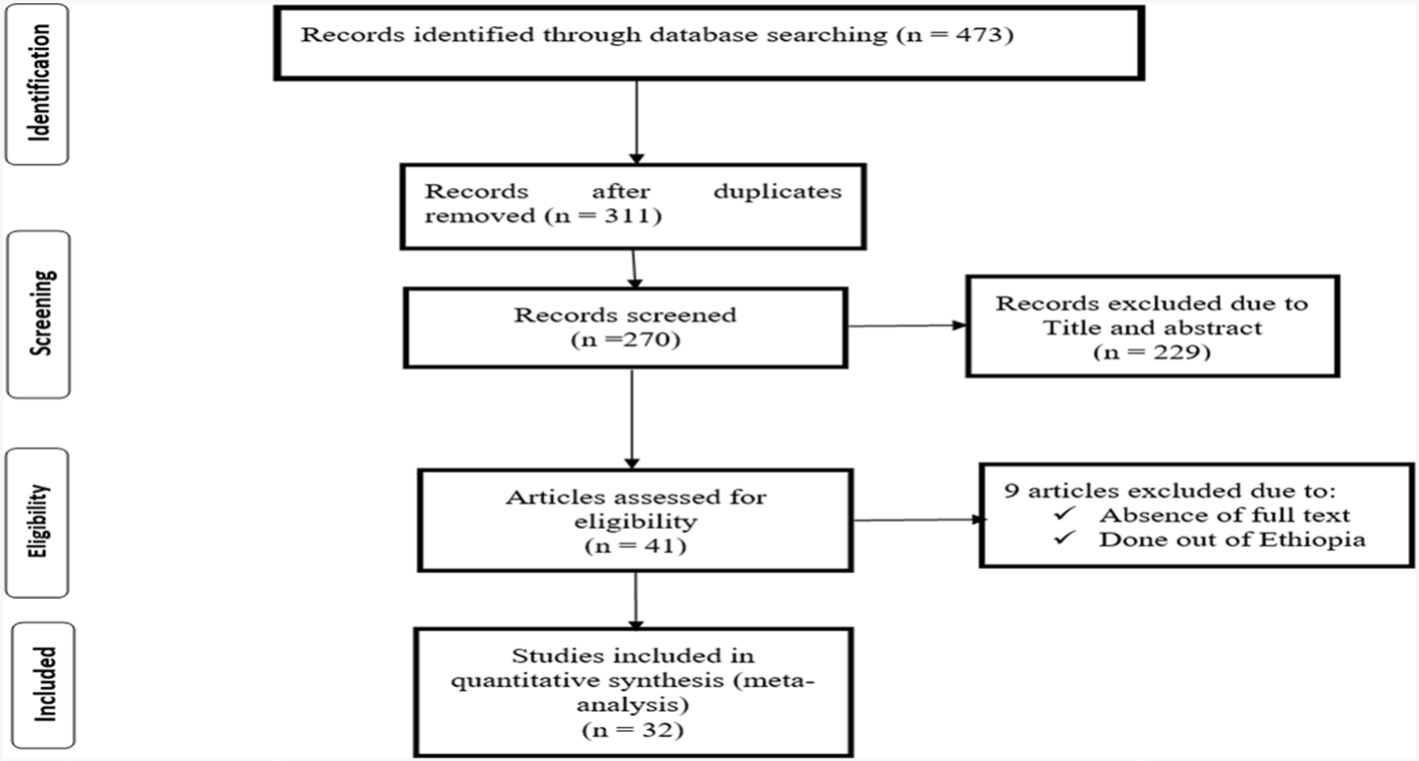
PRISMA flow diagram describing the selection of studies for systematic review and meta-analysis.

A total of 473 articles were identified by searching databases; CINAHL, Google Scholar, Cochrane Library, PubMed, HINARI, and Global Health. Of this, 162 duplicate files were removed using endnote reference manager, 270 articles were dropped due to their titles and abstract and the remaining 41 articles were critically appraised based on the inclusion and exclusion criteria. Finally, 32 full-text articles were included in the systematic review and meta-analysis (Fig. 1).

### Characteristics of the included studies

A total of thirty-two cross-sectional studies involving 18, 354 study subjects were included to estimate the pooled prevalence of premarital sexual practice and its association with peer pressure and watching pornography among young individuals in Ethiopia. Regarding the geographic area, thirteen studies were from Oromia region^14,26,29,34,38,40–43,45,47,51,52^, seven studies were from Amhara region^25,32,33,35–37,50^, five studies were from Tigray region^13,27,48,49,53^, six studies were from SNNPR^5,28,30,39,44,46^ and the rest one study was from Addis Ababa, capital city of Ethiopia^31^. The sample size ranged from 230 students among a study done in Alamata, Tigray region^27^ to 2,766 students among a study done in Eastern Ethiopia, Oromia region^38^ (Table 1). Regarding the sex of the student, twenty-nine studies were conducted among students of both sexes^5,13,14,25–27,30–32,34–53^ and the remaining three studies were done among female students only^28,29,33^.

**Table 1:** Descriptive summary of thirty-two studies included estimating the pooled prevalence of premarital sexual practice and associated factors among students in Ethiopia, 2021.

Authors	Publication year	Region	Study Area	Sample size	Response rate	Prevalence (%)	Quality score
Kasahun et al.^13^	2019	Tigray	Adigrat	572	99.1	17.5	77.8%
Manale et al.^26^	2019	Oromia	Alage	355	97.8	53	66.7%
Abay et al.^27^	2016	Tigray	Alamata	230	100	43.9	66.7%
Sorato et al.^30^	2017	SNNPR	Arba Minch	575	100	43.1	77.8%
Dagim et al.^31^	–	Addis Ababa	Ayer Tena	378	96.9	23.3	66.7%
Bekele et al.^32^	2017	Amhara	Bahir Dar	344	97.7	23.3	66.7%
Tololu et al.^34^	2017	Oromia	Bale Robe	410	97	49	66.7%
Akibu et al.^35^	2017	Amhara	DebreBerhan	604	96	54.3	77.8%
Arega et al.^36^	2019	Amhara	Debretabor	480	96.6	22.5	66.7%
Behulu et al.^37^	2019	Amhara	Debre-Markos	600	96.1	31.3	77.8%
AlemuEarsidoAddila et al.^39^	2020	SNNPR	Hossana	576	95	31.4	77.8%
Girma et al.^42^	–	Oromia	Jimma	523	98.3	21	77.8%
Taye and Asmare^43^	2016	Oromia	Jimma	352	92.5	25.3	55.6%
Gebeyehu et al.^44^	–	SNNPR	Kaffa	410	99.6	39.5	77.8%
Meleko et al.^46^	2017	SNNPR	MizanAman	302	94.9	25.2	66.7%
Girmay et al.^48^	–	Tigray	Northern Ethiopia	560	99.8	21.6	77.8%
Gebreyesus et al.^49^	2019	Tigray	Shire	536	100	47.6	88.9%
Tesfaye et al.^52^	2016	Oromia	Wollega	704	100	30	88.9%
Habte et al.^25^	2018	Amhara	Addis Zemen	276	97.2	32.6	55.6%
Tekletsadik et al.^28^	2014	SNNPR	Aletawondo	394	98.3	18.3	66.7%
Abdissa et al.^29^	2017	Oromia	Ambo	650	92.6	25.7	66.7%
Mulugeta and Berhane^33^	2014	Amhara	Bahir Dar	1093	97.3	30.8	88.9%
Oljira et al.^38^	2012	Oromia	Eastern Ethiopia	2766	96	24.8	88.9%
Biratu et al.^40^	–	Oromia	Jima Arjo	312	98.4	24.4	66.7%
Hurissa et al.^41^	–	Oromia	Jimma	358	99.2	39.7	66.7%
Teferra et al.^45^	2015	Oromia	Bale Goba	302	93.2	42.7	55.6%
ZemenuMengistie et al.^5^	2015	SNNPR	MizanTepi	372	100	35.7	66.7%
Seme et al.^47^	2008	Oromia	Nekemte	676	93.6	21.4	77.8%
Bogale and Seme^50^	2014	Amhara	Shendi	826	97.1	19	88.9%
Endazenaw et al.^51^	2015	Oromia	West Shoa	828	98.2	61.1	88.9%
Ejigu et al.^53^	2012	Tigray	Wukro	588	100	25.9	88.9%
Beyene et al.^14^	2014	Oromia	Yabello	402	95	71.9	66.7%

SSNPR-Southern Nations, Nationalities, and Peoples Region.

### Prevalence of pre-marital sex in Ethiopia

**Figure 2:**
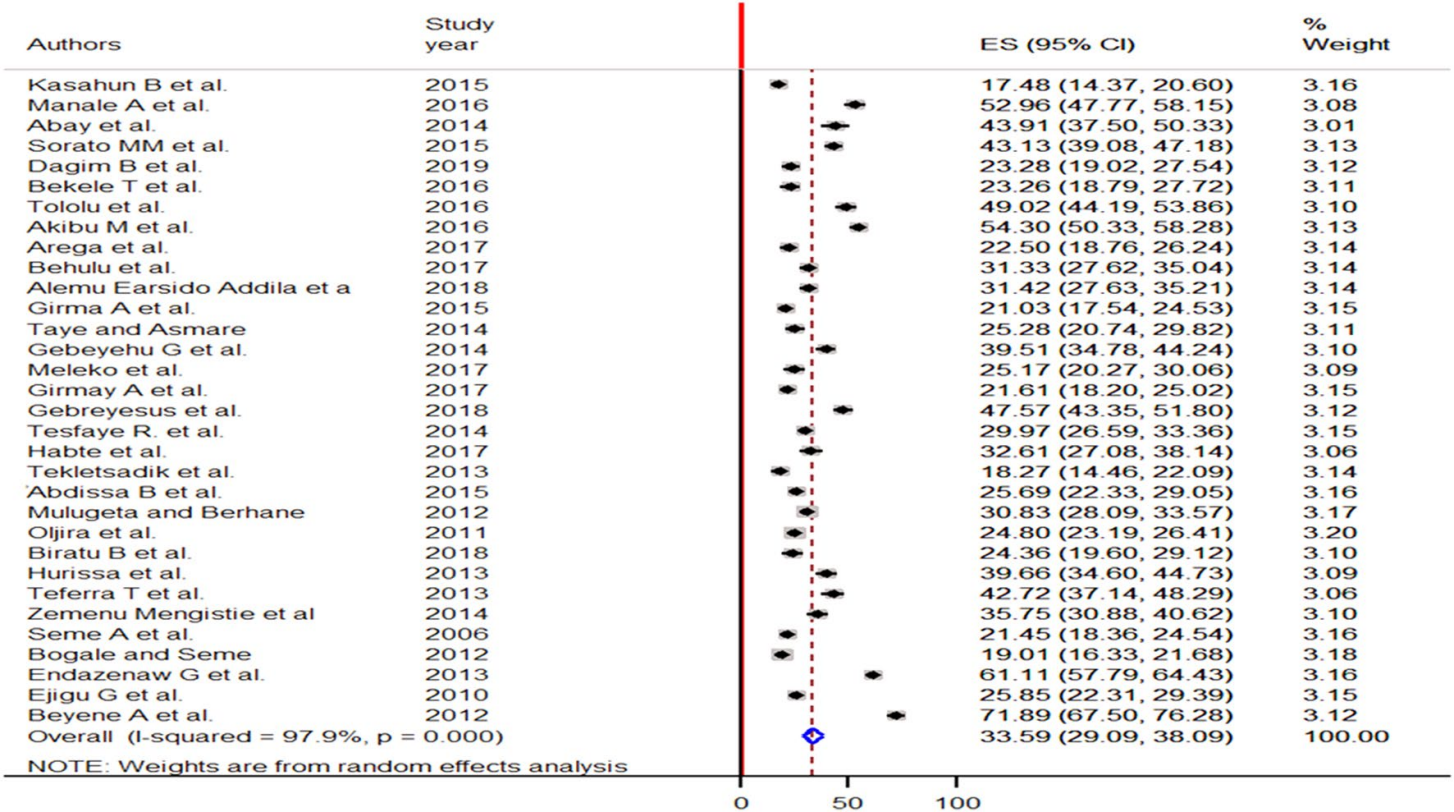
Forest plot of the pooled prevalence of pre-marital sex among young individuals in Ethiopia, 2021.

The result of 32 studies indicated that the pooled prevalence of pre-marital sex among young individuals in Ethiopia was 33.59% (95% CI: 29.09, 38.09). A random-effect model was employed to estimate the pooled effect due to significant heterogeneity across the included studies (I^2^ = 97.9%, *p* = 0.000) (Fig. 2).

**Figure 3:**
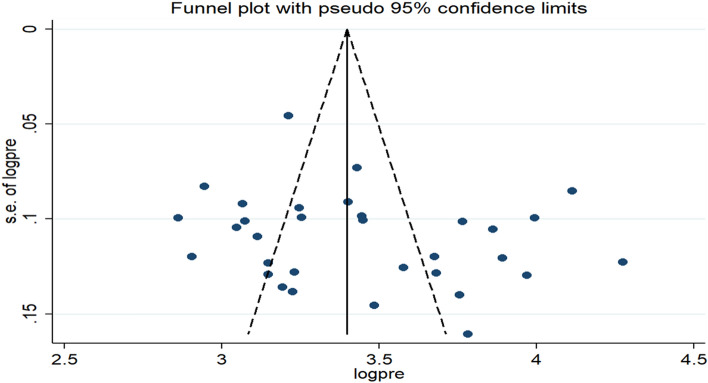
Funnel plot of the pooled prevalence of pre-marital sex among young individuals in Ethiopia, 2021.

The funnel plot and eggers test were used to assess the presence of publication bias. In the funnel plot, effect estimates were distributed asymmetrically which is a sign of the presence of publication bias (Fig. 3). However, the result of the eggers test indicated the absence of statistically significant publication bias (*P* = 0.155).

### Subgroup analysis and meta-regression

To identify the source of heterogeneity among the included studies, subgroup analysis based on the regions where the studies were conducted, sex of the student, residence, study setting, sample size, and the quality score was performed. Although the heterogeneity among the included articles was not resolved, the prevalence of premarital sex was significantly higher among studies done in the Oromia region [37.6%, 95% CI: (28.9, 46.3)] compared to studies conducted in Addis Ababa and Tigray region [29.8, 95% CI: (20.6, 39). Similarly, the prevalence of premarital sexual practice varied significantly among studies conducted in refugee camps and in the community [59.7, 95% CI: (35.9, 83.5)] compared to studies conducted in secondary and preparatory school [27.3, 95% CI: (23.2, 31.5)] (Table 2).

**Table 2:** Subgroup prevalence of pre-marital sex among students in Ethiopia, 2021 (n = 32).

Variables	Characteristics	Included studies	Estimate (95% CI)	I^2^
**Region**	Oromia	13	37.6 (28.9, 46.3)	98.7%
	SNNPR	6	32.2 (24.4, 39.9)	94.9%
	Amhara	7	30.5 (22, 38.9)	97.4%
	Others^a^	6	29.8 (20.6, 39)	97.0%
**Residence**	Urban and rural	20	30.8 (26.3, 35.2)	97.0%
	Urban	12	38.3 (28.7, 47.9)	98.4%
**Sample size**	< 500	16	35.6 (28.2, 43)	97.5%
	≥ 500	16	31.6 (25.8, 37.5)	98.2%
**Sex**	Male and female	29	34.5 (29.5, 39.5)	98.0%
	Female	3	25 (17.9, 32.1)	92.8%
**Study setting**	High school	11	28.1 (21.1, 35)	98.2%
	Secondary and preparatory	3	27.3 (23.2, 31.5)	69.7%
	Preparatory	5	29.1 (22.5, 35.7)	90.9%
	College	5	37.6 (25.2, 49.9)	97.1%
	University	6	38.5 (29.6, 47.5)	96.6%
	Others^b^	2	59.7 (35.9, 83.5)	98.4%
**Quality score**	< 73.6	16	34.7 (27.4, 42)	97.5%
	≥ 73.6	16	32.5 (26.5, 38.5)	98.2%

SNNPR-Southern Nation Nationalities and Peoples Region; ^a^Tigray and Addis Ababa, ^b^community and refugee camps.

Univariate meta-regression analysis was also carried out using study year, response rate, and sample size as a predictor variable. However, neither of them found to be a statistically significant source of heterogeneity among the included studies (Table 3).

**Table 3:** Univariate meta-regression analysis to identify factors associated with the heterogeneity of the prevalence of premarital sex in Ethiopia, 2021.

Variables	Coefficient	*P* value
Sample size	− 0.0047862	0.443
Study year	− 0.2877191	0.777
Response rate	0.2320351	0.837

### The association between peer pressure and premarital sexual practice

**Figure 4:**
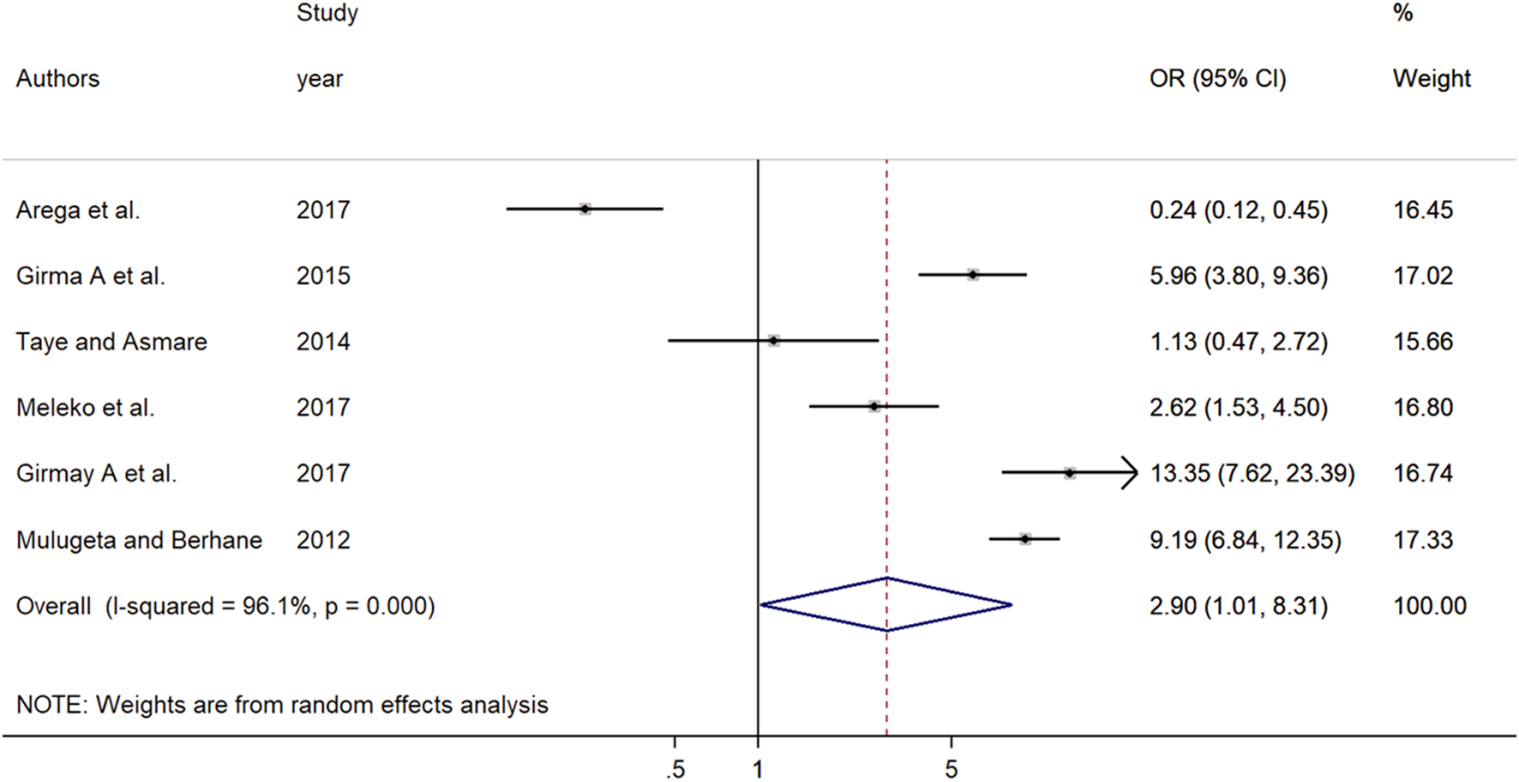
Forest plot of the association between premarital sexual practice and peer pressure among young individuals in Ethiopia, 2021.

The association between peer pressure and premarital sexual practice was estimated based on the results of six studies^33,36,42,43,46,48^. The result showed that young individuals who experienced peer pressure were three times more likely to practice premarital sex compared to their counterparts [OR = 2.90, 95% CI (1.01, 8.31)]. DerSimonian and Laird random-effects model was used to examine the association due to severe heterogeneity among the included studies (I^2^ = 96.1%, *p* = 0.000) (Fig. 4). Publication bias was assessed by using the eggers test and the result of which showed the absence of significant publication bias (*P* = 0.140).

### The association between watching pornography and premarital sexual practice

**Figure 5:**
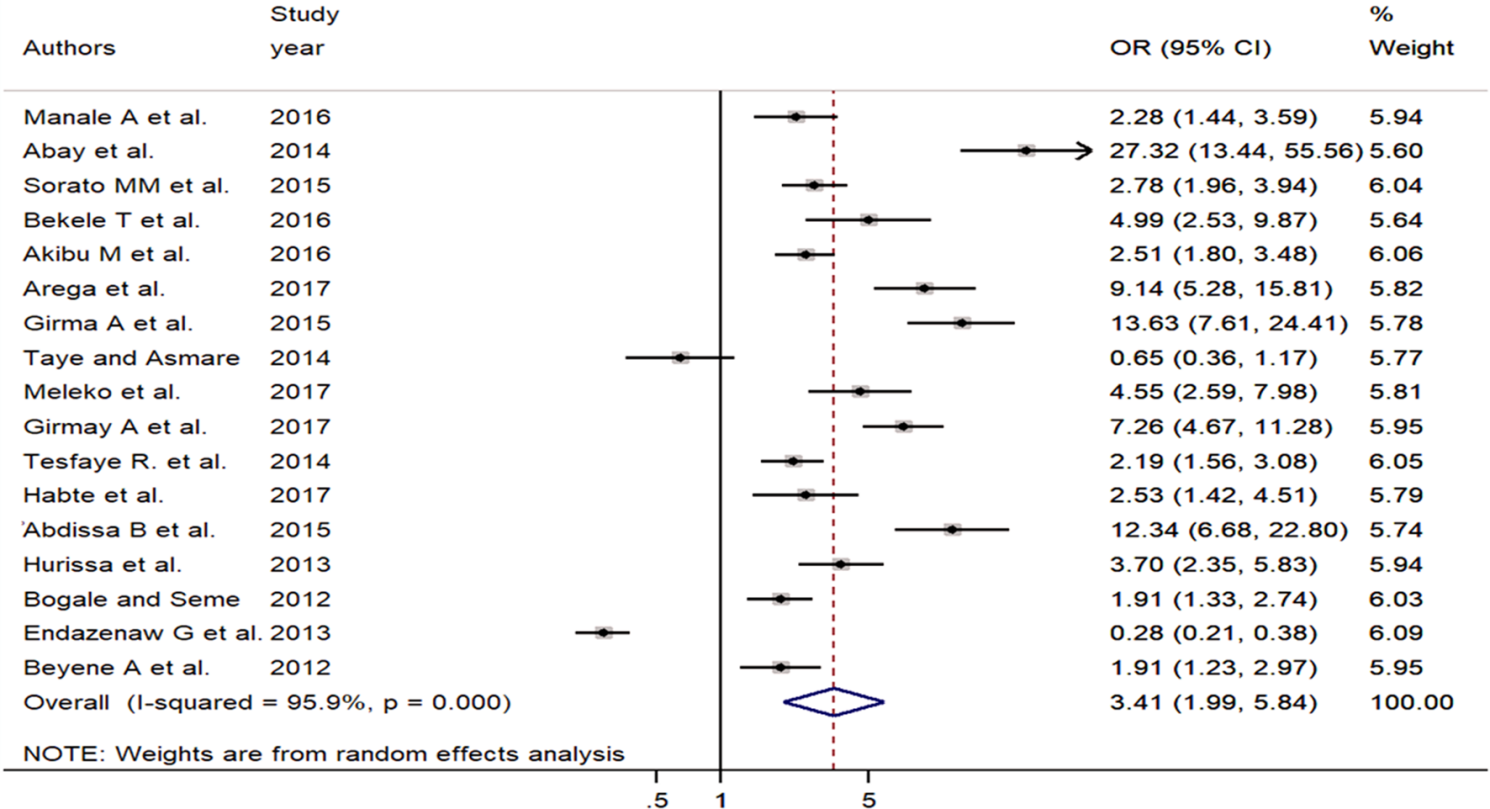
Forest plot of the association between premarital sexual practice and watching pornography among students in Ethiopia, 2021.

A total of seventeen studies were used to assess the association between premarital sexual practice and watching pornography^14,25–27,29,30,32,35,36,41–43,46,48,50–52^. The random-effect meta-analysis evidenced that the odds of practicing premarital sex was 3.4 times higher among young individuals who watched pornography (sex movies) compared to students who didn’t watch sex movies[OR = 3.41, 95% CI (1.99, 5.84)]. Significant heterogeneity was observed among the included articles (I^2^ = 95.9%, *p* = 0.000) (Fig. 5).

**Figure 6:**
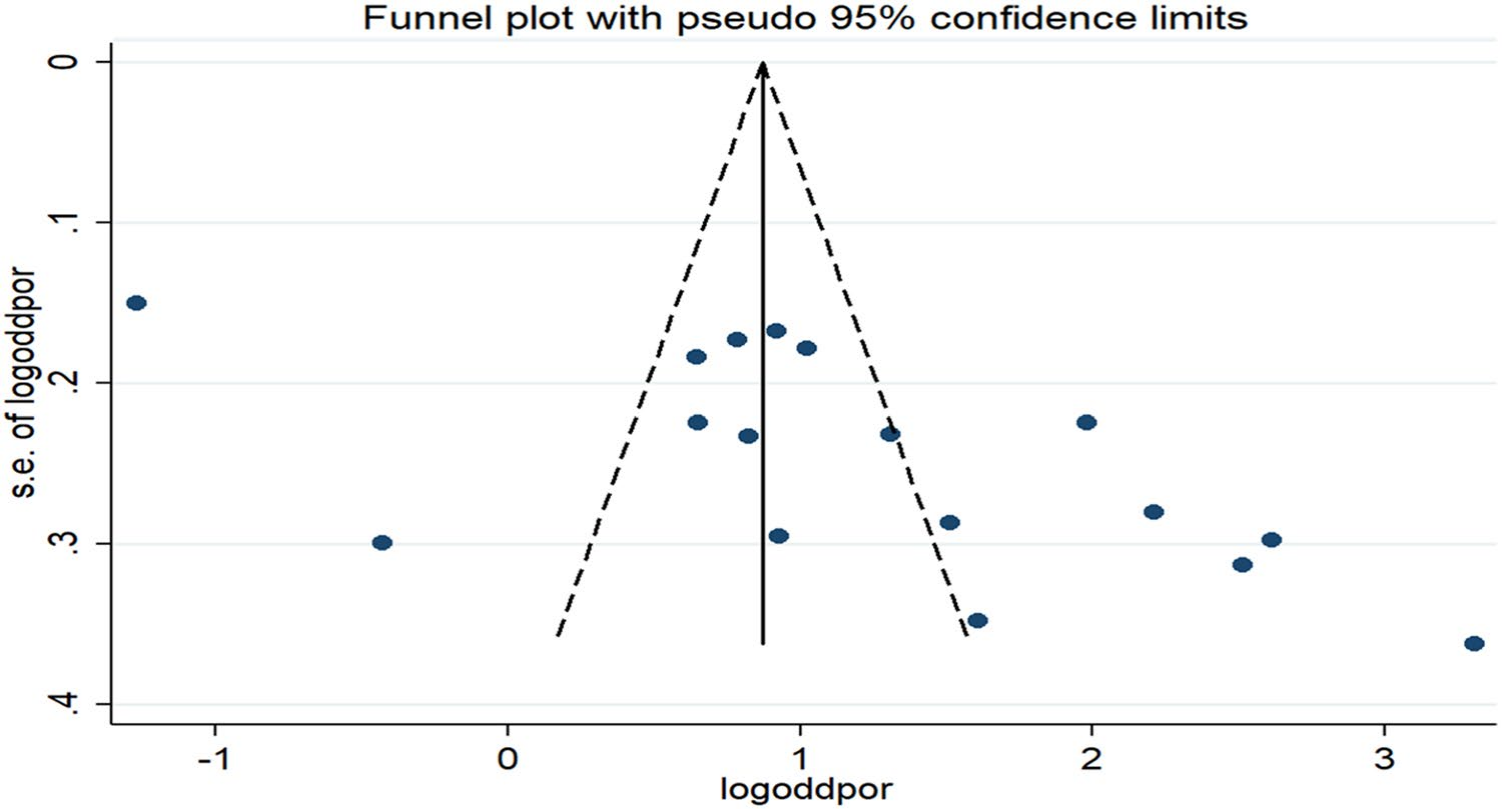
Funnel plot of the pooled odds ratio of watching pornography among young Ethiopia, 2021.

The presence of publication bias was checked by using both funnel plot and eggers test. The result showed that there was an asymmetrical distribution of the individual studies on the funnel plot, indicating the presence of publication bias (Fig. 6). The Egger tests statistics also revealed the presence of statistically significant publication bias (*P* = 0.005).

**Figure 7:**
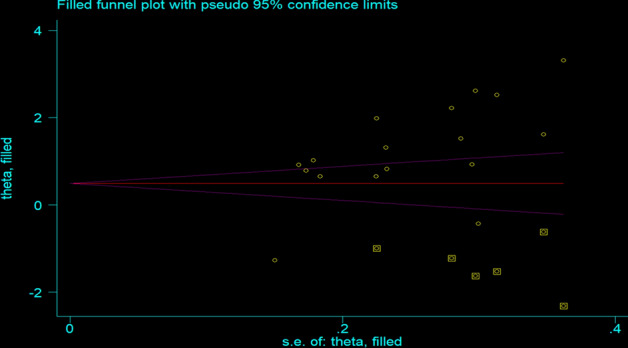
The funnel plot of a simulated meta-analysis containing 17 studies.

Duval and Tweedie’s 'trim and fill' analysis was conducted to adjust the effect of publication bias among studies included to determine the association between premarital sex and watching pornography^62^, and there was significant variation in the newly estimated pooled odds ratio (the adjusted point estimate) [OR = 1.23, 95% CI: (0.69, 1.76)] as compared to the initial or observed point estimate[OR = 3.41, 95% CI: (1.99, 5.84)] (Fig. 7).

## Discussion

In this study, the pooled prevalence of pre-marital sex among young individuals in Ethiopia was 33.6% (95% CI: 29.09, 38.09). Peer pressure had a statistically significant association with premarital sex. However, as the trim-and-fill adjusted OR indicated, there was no significant association between watching pornography and premarital sex.

The pooled prevalence of premarital sex was comparable with studies conducted in Bangladesh (30%)^24^and Vientiane, Lao PDR (34%)^22^. But, it was higher than studies conducted in Ebonyi State, Nigeria(27.6%)^63^, Katmandu, Nepal(19.6%)^64^, Malaysia(5.4%)^65^, and Vietnam (16.9%)^66^. However, the finding is lower than a study conducted in Beijing, China (41%)^67^ and another study conducted in Nepal (39%)^23^. The possible justification for the discrepancy could be due to the differences in the study population, study settings, and educational, cultural, socioeconomic, and lifestyle differences across countries. In addition, the difference in the research methodology (study design and data collection method used), the difference in the adolescent reproductive health care policy, reproductive health service coverage, and utilization across countries could be responsible for the variation.

In this meta-analysis, peer pressure was found to increase the likelihood of engaging in pre-marital sex similar to a study conducted in Anambra state of Nigeria^68^, University of Maiduguri, Nepal^69^, and Vientiane, Lao PDR^22^. Studies conducted in Singapore^17^, Kaduna state university^70^, and Vietnam^66^ also point out that the odds of pre-marital sexual practice were higher among students with peer influence compared to their counterparts. This could be because peer pressure is one of the most influential factors that determine youth’s sexual behavior. Peer influence is powerful in changing the attitude, behavior, and personality of young individuals. Young individuals usually tend to follow the behavior that their intimate peers practiced^71^. There was a piece of evidence that, peer behavior in all societies is a model for individuals’ behavior, especially in matters of sexuality among youths and adolescents^72^.

As egger's test evidenced, significant publication bias was observed among studies include to determine the association between premarital sex and watching pornography. To adjust the effect of publication bias among the included studies, Duval and Tweedie’s 'trim and fill' analysis was conducted. After doing trim-and-fill analysis, a major discrepancy was observed between the initial or observed point estimate (crude OR) and publication-bias adjusted OR i.e. the crude OR shows the presence of a statistically significant association between watching pornography and premarital sex whereas the adjusted OR indicates the absence of significant association between watching pornography and premarital sex.

As a limitation, only four among the nine regions were represented in this meta-analysis. The study was also limited to articles published only in the English language. Heterogeneity was observed in all analyses although we performed meta-regression and subgroup analyses. Moreover, all of the articles included in this meta-analysis were cross-sectional and had a small sample size, and thus might affect the pooled estimates.

## Conclusions

The proportion of premarital sexual practice among young individuals in Ethiopia was considerably high. Peer pressure had a statistically significant association with premarital sex. However, the trim-and-fill adjusted OR indicates the absence of a significant association between watching pornography and premarital sex. The national government should design and implement adolescents and youth-sensitive sexual and reproductive health policies and strategies to tackle premarital sexual practice and its consequences. Educational institutions should incorporate skill-building programs in the curriculum and design and implement peer-to-peer counseling services to cope with peer influence. Sex education and behavioral change communications should also be strengthened to address factors associated with pre-marital sexual practices.

## Supplementary Information

Supplementary Information 1.

Supplementary Information 2.

Supplementary Information 3.

## Data Availability

The datasets used and/or analyzed during this study are available from the corresponding author on reasonable request.

## Abbreviations

HIV/AIDS: Human immune virus/acquired immunodeficiency syndrome
JBI: Joana Brigg's Institute
PRISMA: Preferred reporting items for systematic reviews and meta-analysis
SDG: Sustainable development goal
SSA: Sub-Saharan African
STI: Sexual transmitted infections
WHO: World Health Organization
