# Supplementary material for: Pre-marital sex and its association with peer pressure and watching pornography among young individuals in Ethiopia: a systematic review and meta-analysis
Source: Sci Rep. 2022 Jun 10;12:9572. doi: 10.1038/s41598-022-13448-y (PMC9427853; doi:10.1038/s41598-022-13448-y)
Supplement: Supplementary file 2 — Supplementary Information 2. [file 41598_2022_13448_MOESM2_ESM.docx]

**Supplementary file 2:** Search strategy used to estimate the pooled prevalence of pre-marital sex and its association with peer pressure and watching pornography among young individuals in Ethiopia.

PubMed

***(((((((prevalence) OR (proportion)) OR (magnitude)) OR (incidence)) AND ((((“premarital sex”) OR (“premarital sexual intercourse”)) OR (“premarital sexual debut”)) OR (“premarital sexual practice”))) AND (((((factors) OR (predictors)) OR (determinants)) OR (“risk factors”)) OR (“associated factors”))) AND ((((young) OR (youths)) OR (students)) OR(adolescents))) AND (Ethiopia) Filters applied: Humans, from 2000/1/ 1- 2021/3/31***

*HINARI*

((proportion) OR (magnitude) OR (prevalence) OR (incidence)) AND (("premarital sex") OR ("premarital sexual intercourse") OR ("premarital sexual debut") OR ("premarital sexual practice")) AND ((factors) OR (determinants) OR ("associated factors") OR ("risk factors")) AND ((student) OR (youths) OR (young) OR (adolescents)) AND (Ethiopia)

Filters applied: Humans, from 1/1/2000- 3/31/2021
